# Ethical arguments against coercing provider participation in MAiD (medical assistance in dying) in Ontario, Canada

**DOI:** 10.1186/s12910-020-00486-2

**Published:** 2020-06-03

**Authors:** Travis Carpenter, Lucas Vivas

**Affiliations:** 1 Unity Health Toronto, Toronto, Ontario Canada; 2 Department of Medicine, University of Toronto, 30 The Queensway, Toronto, Ontario M6R 1B5 Canada; 3 William Osler Health System, Brampton, Canada

**Keywords:** Medical assistance in dying, Euthanasia, Assisted dying, Right to die, Conscientious objection, Conscience rights

## Abstract

It has historically been a crime in Canada to provide assistance to someone in ending their own life, however, this paradigm was inverted in 2015 when the Supreme Court of Canada (SCC) ruled that restrictions on this practice, within certain defined parameters, violated the right to life, liberty, and security of the person. Subsequently, recent legal and policy decisions have highlighted the issue of how to balance the rights of individuals to access MAiD with the rights of care providers to exercise conscience-based objections to participation in this process. We argue that there is significant harm and ethical hazard in disregarding individual and institutional rights to conscientious objection and since measures less coercive than the threat of regulatory or economic sanctions do exist, there should be no justification for such threats in Canada’s health care systems.

## Background

As in most Western societies, it has historically been a crime in Canada to provide assistance to someone in ending their own life. This paradigm was inverted in 2015 when the Supreme Court of Canada (SCC) ruled that restrictions on this practice, within certain defined parameters, violated the right to life, liberty, and security of the person articulated in the Canadian Charter of Rights and Freedoms [1].^1^ This legal change resulted from extensive public debate and disagreement over many of the profound ethical and practical implications of euthanasia. The practice known generally as voluntary active euthanasia has now been codified into Canadian law as “medical assistance in dying”, or “MAiD”.

Ethical disagreement about the nature of MAiD has not been settled by the Supreme Court ruling or the subsequent legislative and policy responses. Some view MAiD as a commendable act of compassion, while others consider it an act of severe violence (indeed, an act still condemned by the Criminal Code of Canada except under the specific conditions of MAiD [1, 2]). A related issue (although equally contentious) is the question of how to balance the rights of individuals to access MAiD with the rights of care providers to exercise conscience-based objections to participation in this process. As governments and regulators attempted to set rules for MAiD provision, tensions flared in the province of Ontario over two predominant issues: regulatory bodies threatening severe punishment for individual healthcare providers who do not initiate “effective” referrals for MAiD, and pressure to compel faith-based (but publicly-funded) institutions to facilitate MAiD [3–5]. This paper will argue there is significant harm and ethical hazard in disregarding individual and institutional rights to conscientious objection. The onus is on government and regulators to demonstrate why coercive measures are needed to provide appropriate access to MAiD. Less coercive measures, such as centralized care coordination systems, allow governments to achieve the goal of making MAiD available without directly involving individuals in a causal chain that involves them in MAiD. The existence of these systems render any coercive measures directed to individual providers or institutions unjustifiable. Other jurisdictions considering legalization of euthanasia should consider the current state of affairs in Ontario if they wish to respect the fundamental rights of health care workers.

## Main text

Health care providers may object to participation in MAiD to various degrees. Some decline to participate in the actual act of euthanasia but provide assessments and support for the same. Others do not wish to be involved in assessments or any ancillary participation. A minority does not wish to provide direct referrals (the issue at question in the Ontario court case described above). For the purposes of this discussion, any limitation in participation in MAiD, if it arises out of a judgement of conscience (consistent and rational), would be considered a conscientious objection.

The SCC confirmed in its initial MAiD ruling that any regulatory or legislative response to its decision must reconcile and respect rights of both patients and physicians [2]. Clinicians have individual rights to freedom of religion and conscience under the Canadian Charter – indeed, these are enumerated among the fundamental rights that underpin the Charter’s structure. In a separate case from 2015, the SCC established that institutions and organizations can have a Charter right to freedom of religion [6]. Presumably, given the holistic nature of Charter rights, this also extends to a freedom of conscience and conscientious objections. Insofar as refusal to participate in the legal MAiD regime is a rational exercise of religious or conscientious convictions, this right of refusal must be balanced with the freedom to obtain assistance in suicide.

There are a number of moral analyses, distinct from the legal analysis, that also support the need to balance rights of clinicians, institutions, and those seeking to end their lives. A libertarian analysis suggests that just as someone may have a fundamental right to die, physicians and faith-based hospitals have an equally-fundamental right to govern their own actions, including exercising conscientious objections to MAiD. This strict interpretation of self-ownership would indicate it is wrong to sacrifice the rights of one for the benefit of another [7] and therefore governments and regulators should not be able to compel providers to complete or facilitate MAID against their will. Even if a right to die is established as a cultural norm, it can be argued that coercive force of law should not be used to promote desired virtues or the moral convictions of the majority [7].

A different perspective on conscientious objection to MAiD arises from the Kantian categorical imperative in its second formulation: treating human beings as ends and not only means [7]. Health care providers are not only means to the delivery of services, but valuable moral agents in their own right. It is immoral to compel physicians to act against their deeply-held beliefs, particularly if it can be demonstrated that these beliefs stem from rational objections to MAiD and not merely from distaste or animus. Conscientious objections to MAiD are usually grounded in rational arguments about the meaning and ends of medicine: helping the sick, doing no harm, and respecting the traditional Hippocratic principles encapsulated in the specific pledge to “neither give a deadly drug to anybody who asked for it, nor … make a suggestion to this effect” [8]. Respect for objections grounded in such reasons is mandated by the categorical imperative. This is true even if the consequence of a conscientious refusal is a delay or barrier to accessing MAiD, since clinicians are not merely instruments to be used in order to achieve system goals.

A criticism of a focus on provider conscience rights is that supporting such rights violates the harm principle. It is argued that conscientious objections to MAiD unfairly harm dying patients, unjustly depriving them of appropriate access to an essential and medically-indicated service. As a response to this criticism, proponents of conscientious objection should acknowledge that respect for their own freedom of conscience does not entitle them to “run interference” on the liberties of dying patients, especially in a pluralistic society [7, 9]. Systems must exist to ensure that options for MAiD are not hidden from patients and that providers cannot unduly obstruct a patient’s exercise of legally-available liberties. To protect patients, regulators and government have a responsibility to publicize MAiD adequately, and provide an alternative means of access (electronic or otherwise) to bypass non-participating providers [10]. It may also be conceivable to create registries of participating or objecting providers, although this option would need to account for the significant threat of harm to providers (whatever their stance) and the invasion of the professional privacy of clinicians. One example of a non-coercive system is found in the Canadian province of Alberta: a Central Coordination Service (CCS) facilitates referrals for MAiD without requiring clinician participation. This system has a low barrier to access, making it accessible to patients as well as clinicians. As it is not directly a MAiD-providing service but rather a “referral clearinghouse”, conscientious objectors (individual and institutional) in Alberta have not objected to interacting with the CCS. Nevertheless, this approach to rights-balancing may be perceived as inadequate by those who assert that dying patients are vulnerable and may not be able to access MAiD if forced to seek alternative pathways. A pragmatic assessment of the real-life barriers to MAiD is outside the scope of this ethical commentary, although legal-regulatory concerns seem to be the major barrier [11], not conscientious objections. An approach like the CCS does, however, seriously try to address the objection based on harm to patients while respecting conscience rights of providers.

A more fundamental criticism of conscientious objection is that, even if a patient’s access to services is unimpeded, it is contrary to public reason to allow physicians in a publicly-funded system to choose which lawful procedures they will provide [9, 12]. The patient and the service should be at the center of an encounter, not the provider. Doctors should be required to provide all lawful services that a patient medically qualifies for and should not impose moral judgements at the bedside [9, 12]. The Kantian categorical imperative is felt not to apply, since a career in medicine is a choice and individuals should not enter the profession if they are not willing to satisfy the requirements.

This argument devalues medicine as a profession and parallels the unfortunate cultural trend of treating physicians like technicians merely trained and paid to do a job. Irish philosopher and ethicist Christopher Cowley argues “medicine is not a normal job, and demands so much more from practitioners than merely fulfilling a contract. The best doctors are those who identify with the role and who are motivated to go beyond their contractual duties … and who have an understanding of a job well done that is separate from remuneration and promotion.” [9] Physician and ethicist Edmund Pellegrino likewise argued that medicine, along with other professions, serves the common good most completely when it actively defines the moral commitments its members make, rather than simply responding to societal demands [13]. Both of these thinkers – along with most physicians – propose that physicians themselves must be active participants in the ethical debates that shape our common public morality. Many conscientious objectors adhere to moral codes that extend far beyond the question of MAiD, which provide moral arguments for other professional virtues such as selflessness and devotion to service over financial or other rewards. The existence of these codes strengthens the ability of the profession of medicine to define its moral commitments, even if it sometimes leads to conscientious objections. The fact that many of these codes are grounded in religion makes some skeptical of whether these individuals practice better medicine [12], but such judgements are more indicative of pre-existing biases about religion and religious persons. Whether conscientious objections arise out of religious or philosophical considerations, however, does not change the fact that such objections reflect a valuable addition to the moral discourse within the profession and therefore to the profession’s contribution to the common good. Furthermore, compelling existing physicians to adhere to new rules that assault their deepest convictions will (at best) lead to demoralization and burnout; at worst, it could force them out of the profession, depriving the system of both valuable providers and principled mentors.

From this latter concern inevitably follows the consequentialist concern that enforcing restrictions on conscience rights may paradoxically result in worse service provision to dying patients. A majority of palliative care physicians in Canada have vocally opposed MAiD and have advocated that for many (but clearly not all), a desire to pursue MAiD stems from suffering and fear in an environment where there is inadequate provision of high-quality palliative care [14, 15]. Furthermore, individual palliative care providers (already in short supply) may stop accepting patients if they fear they may be compelled to violate their conscience rights [3], resulting in decreased overall provision of services. Similarly, faith-based institutions (especially Catholic hospitals and hospices) are motivated and likely irreplaceable providers of palliative care in Ontario and elsewhere [4, 16]. They are often more likely to serve vulnerable populations and may even provide higher-quality and more patient-centered clinical care [17, 18]. Sanctioning these providers financially or otherwise would be a Pyrrhic victory for MAiD proponents that would almost certainly do more harm than good [4].

Some may argue that, while individual conscientious objection may be allowed, institutional exemptions should still not be accommodated. Faith-based institutions (which in Canada are primarily Catholic) provide publicly-funded services to all citizens regardless of religious affiliation, and therefore they may not meet the established Supreme Court requirements for institutional protection as they do not serve a primarily religious purpose [6]. In many cases, a majority of employees, contractors and patients are not Catholic. As “bricks and mortar have no conscience”, government could still mandate Catholic hospitals to provide MAiD [5]. However, although publically-funded for provision of services, these hospitals are independent entities with statements of mission, vision and values that are not government-imposed. Institutional independence allows them to be a valuable moral bulwark against government encroachments, a part of what Henry Mintzberg calls the “plural sector” [19] of society (neither public nor private). In the case of faith-based health facilities, the mission is grounded in a religious community of belief and practice, and in order for these institutions to maintain organizational integrity, they must be allowed to live out these beliefs. Practically, faith-based institutions have legal ownership of their facilities, and it would constitute an incredibly poor use of billions of scarce healthcare dollars to purchase, rent, or otherwise expropriate these facilities [5]. An additional argument against institutional exemptions is that in small communities, faith-based institutions may be the sole providers of care and patients would be unjustly hindered in seeking MAiID. This argument is insufficient in the context of existing clinical realities and disparities. Provision of routine clinical care (including necessary end-of-life interventions like palliative biliary stent insertion or even hospice placement) is often not possible in small communities, and hundreds of patient transfers are completed daily for this purpose. There are already huge disparities between care available in small communities compared to larger centres, so it is unclear why MAiID would require implementation of coercive measures when other more profound and harmful inequities remain unaddressed. Additionally, MAiD need not be completed in hospital and could be completed in patient’s homes, nursing facilities, or by mobile teams [20].

## Conclusions

In Ontario’s context, coercion of individuals or institutions to participate in MAiD should be considered as unethical and unjustified. As discussed above, the importance of individual and institutional conscience is defensible from multiple lines of ethical and practical argumentation, and people coming from a variety of ethical stances can agree that a free conscience is a net benefit to the health care system. Instead of disregarding fundamental rights, the government of Ontario and the CPSO should establish and publicize a transparent and easily-available registry of healthcare providers and institutions that deliver MAiD [5, 10]. Requiring providers to inform patients of the existence of such a registry would be a far more minimal impairment of conscience rights than mandating referral [10]. Providers who act in bad faith to misinform about MAiD should be appropriately disciplined. The registry should clearly define how patients can easily access MAiD resources, and Ontario should emulate Alberta which has hired dedicated nurse professionals to field inquiries, initiate referrals, and navigate regulatory requirements on behalf of patients [21]. As discussed, both proponents and opponents of conscience rights could likely agree that the current status quo is inadequate and that the government should allocate appropriate resources to both MAiD and especially to high-quality palliative care. Careful planning should consider appropriate locations and settings for services, including protocols for facilitating assessment and treatment of patients at sites which do not offer MAiD.

These solutions constitute a reasonable way to respect and preserve fundamental rights and freedoms for all in our pluralistic society. While dying patients should be able to exercise self-determination and a right to die, simultaneously accommodating conscientious objection does not impose undue harms and is an ethical necessity.

## Data Availability

Not applicable.

## Abbreviations

CCS: Central Coordination Service
CPSO: College of Physicians and Surgeons of Ontario
MAiD: Medical Assistance in Dying
SCC: Supreme Court of Canada

## Footnotes

1 The Charter of Rights and Freedoms is a set of guarantees enshrined in the 1982 Constitution
